# Evaluating treatment outcomes stratified by regimen among drug-resistant TB patients in Sierra Leone

**DOI:** 10.5588/pha.25.0056

**Published:** 2026-03-06

**Authors:** J.A. Koroma, B.D. Fofanah, D. Nair, E.M. Kamau, I.F. Kamara, M.A. Sesay, I.S. Turay, N. Sesay, F. Kanu, W.K. Lahai, J.S. Kanu, A.T. Koroma, F. Fornah, A.L. Seisay, S.S. Bailor, R. Harding, S. Emezue, G.B. Tefera, G. Ameh, M. Mazzi, S. Lakoh, M. Mahmoud

**Affiliations:** 1 Ministry of Health, Freetown, Sierra Leone;; 2 World Health Organization Country Office, Freetown, Sierra Leone;; 3 Independent Researcher, Scottsdale, AZ, USA;; 4 UNICEF, UNDP, World Bank, WHO Special Programme for Research and Training in Tropical Diseases (TDR), Geneva, Switzerland;; 5 Partners in Health, Koidu, Sierra Leone.

**Keywords:** tuberculosis, operational research, BPaL, BPaLM, standardised short regimen, individualised long regimen, SORT IT

## Abstract

**SETTING:**

Sierra Leone has a high burden of drug-resistant TB (DR-TB), managed at three treatment centres.

**OBJECTIVE:**

To compare treatment success between BPaL (bedaquiline, pretomanid, and linezolid)/BPaLM (bedaquiline, pretomanid, linezolid, and moxifloxacin) and the standardised short and the individualised long regimens among DR-TB patients and identify predictors of unsuccessful outcomes.

**DESIGN:**

Retrospective cohort study utilising routinely collected national DR-TB data from January 2022 to December 2024.

**RESULTS:**

Among 598 DR-TB patients registered from 2022 to 2024, 571 with complete outcomes were analysed. Overall treatment success was 80.2%, highest with BPaL/BPaLM (87.1%) compared with the standardised short (78.8%) and individualised long regimens (70.4%). Adjusted analyses showed BPaL/BPaLM remained strongly associated with higher success than the individualised long (adjusted risk ratio [aRR] 2.89; 95% confidence interval [CI] 1.80–4.64) and standardised short regimens (aRR 1.46; 95% CI 1.04–2.05). HIV co-infection and underweight body mass index independently predicted poor outcomes. Findings were consistent across propensity-weighted and sensitivity analyses.

**CONCLUSION:**

Under routine programmatic conditions in Sierra Leone, BPaL/BPaLM achieved higher treatment success than standardised short or individualised long regimens. However, HIV co-infection and undernutrition predicted poorer outcomes, underscoring the need for integrated nutritional support, expanded drug-susceptibility testing, and strengthened TB/HIV services.

TB has regained its position as the leading cause of death from a single infectious agent globally, after COVID-19, despite a partial recovery in diagnosis and treatment following pandemic-related disruptions. The disease continues to affect vulnerable populations disproportionately, exacerbating poverty, inequality, and health system strain.^1,2^ Drug-resistant TB (DR-TB), caused by *Mycobacterium tuberculosis* strains resistant to first-line anti-TB drugs, poses a significant challenge for TB control efforts.^3^ DR-TB is classified by resistance patterns: isoniazid-resistant TB (Hr-TB), rifampicin-resistant TB (RR-TB), multidrug-resistant TB (MDR-TB), pre-extensively drug-resistant TB (pre-XDR-TB), and extensively drug-resistant TB (XDR-TB).^4,5^ According to the 2023 Global TB Report, an estimated 410,000 people developed MDR/RR-TB globally in 2022, with the African region reporting 22.5 cases per 100,000 population. In many African countries, systemic barriers – including limited diagnostic capacity, weak supply chains, and under-resourced health systems – continue to undermine DR-TB management.^6–10^ To address these challenges, the WHO launched the End TB Strategy and, in 2022, updated its treatment guidelines to recommend shorter, safer, all-oral regimens for MDR/RR-TB. The 6- to 9-month BPaLM (bedaquiline, pretomanid, linezolid, and moxifloxacin) and BPaL (bedaquiline, pretomanid, and linezolid) regimens – comprising bedaquiline, pretomanid, linezolid, and moxifloxacin (or without moxifloxacin) – were endorsed for eligible patients, particularly where fluoroquinolone drug-susceptibility testing (DST) is available.^11,12^

Sierra Leone introduced the 20-month individualised long regimen in 2017 and the 9–12-month standardised short regimen in 2020. A national study prior to the BPaLM/BPaL rollout reported a 73% success rate with the standardised short regimen. Since the inception of DR-TB management, no evaluation has yet been conducted to compare outcomes across all available regimens under programmatic conditions. Despite these advances, no national evaluation has compared outcomes across all available DR-TB regimens under routine programmatic conditions. Understanding regimen-specific outcomes is essential for guiding scale-up, strengthening clinical decision-making, and informing policy.

We therefore established this study to compare treatment success rates among DR-TB patients initiated on BPaL/BPaLM versus the standardised short and individualised long regimens. Secondary objectives were to identify predictors of unsuccessful outcomes; compare outcomes across treatment centres; and assess robustness through sensitivity and time-to-event analyses.

## METHODS

We conducted a retrospective comparative cohort study using routinely collected data from all three DR-TB treatment centres in Sierra Leone. Regimen assignment followed national eligibility criteria and was non-random, introducing potential confounding by indication.

### General setting

The Ministry of Health delivers DR-TB services through the National Leprosy and Tuberculosis Control Programme (NLTCP), which implements the 2021–2025 National Strategic Plan aimed at detecting and treating 90% of TB cases by 2025 and reducing TB mortality.^13^

### Specific settings

Sierra Leone’s DR-TB programme began in 2017 at Lakka Government Hospital (tertiary level, 150 beds), followed by Makeni and Kono Government Hospitals (secondary level, 12 and 10 beds). More than 1,100 MDR/RR-TB patients have been treated nationally, with a 73% success rate among cohorts completing treatment in 2021.^2^

### Study population and period

We included all DR-TB patients enrolled between 1 January 2022 and 31 December 2024 across the three centres, grouped by initial regimen. The long (2017), short (2020), and BPaL/BPaLM (2022) regimens were introduced at different times; we restricted to patients starting from 2022 and adjusted for year of initiation to account for calendar-time effects.

### Regimen and eligibility

National guidelines define three eligible all-oral regimens: 1) individualised long regimen: For MDR/RR-TB patients previously exposed to second-line medicines, including >1 month of bedaquiline; 2) standardised short regimen: For MDR/RR-TB patients without prior second-line treatment and with confirmed fluoroquinolone susceptibility; and 3) BPaL/BPaLM (6–9 months): BPaL for rifampicin- and fluoroquinolone-resistant TB (pre-XDR), or BPaLM for RR-TB or MDR-TB with confirmed fluoroquinolone susceptibility.

Allocation was based on DST, prior exposure to second-line medicines, and site-level practice. Differences in regimen introduction dates and casemix across centres created potential confounding by indication and site-level clustering. These were addressed using multivariable adjustment, cluster-robust standard errors, and propensity-score weighting.

### Inclusion and exclusion criteria

We included all patients initiated on BPaL/BPaLM, the standardised shorter regimen, or the individualised longer regimen. We excluded patients still on treatment at data lock (*n* = 18), those without a documented outcome, those without an assigned regimen (*n* = 8), and two patients placed on RHZE*Levofloxacin, which is not an approved DR-TB regimen.

### Data variables and sources

Data were obtained from the national electronic MDR-TB database and facility registers. Variables included demographics (age, sex, region), clinical data (body mass index [BMI], HIV, resistance, prior TB), treatment centre, year of initiation, and initial regimen. Outcomes were successful (cure/completion), unsuccessful (failure/death/loss to follow-up [LTFU]), or not evaluated. BMI was categorised as underweight (<18.5 kg/m^2^), normal (18.5–24.9 kg/m^2^), or overweight/obese (≥25 kg/m^2^).

### Statistical analysis

Analyses used STATA 18. Descriptive statistics and *χ*^2^ tests compared groups. Modified Poisson regression with robust SEs estimated adjusted risk ratios (aRRs) for treatment success, adjusting a priori for age, sex, BMI, HIV, resistance, prior TB, centre, and year (individualised long as reference). Missing data for BMI, prior TB, and HIV status were handled via multiple imputation. Sensitivity analyses included inverse probability of treatment weighting, centre-level clustering, and time-to-event analysis (Kaplan–Meier, Cox regression). Significance was set at *P* ≤ 0.05.

### Ethical statement

Ethical approval for this study was obtained from the Sierra Leone Ethics and Scientific Review Committee (SLESRC 015/02/2025). As the study utilised anonymised secondary programme data, the requirement for informed consent was not deemed necessary, and permission to use the data for this study was obtained from the Chief Medical Officer of the Ministry of Health in Sierra Leone.

## RESULTS

A total of 598 patients with a recorded diagnosis of DR-TB were registered for treatment between January 2022 to December 2024 across the three centres. After applying exclusion criteria (18 patients still on treatment, 6 with undocumented outcomes, 3 with undocumented regimen), 571 patients with complete outcome data formed the primary analysis cohort. The distribution of patients across centres in the full dataset was: Lakka (*n* = 315, 52.7%), Makeni (*n* = 183, 30.6%), and Kono (*n* = 100, 16.7%); this distribution was similar in the analysis cohort (Lakka 301, Makeni 174, and Kono 96). The median age of patients was 39 years (interquartile range [IQR] 28–52), and 403 (70.6%) were male. The distribution of treatment regimens was: BPaL/BPaLM 292 patients (48.8% of full cohort, 51.1% of analysis cohort), standardised short regimen 118 (19.7%, 20.7%), and individualised long regimen 188 (31.4%, 32.9%). Regimen allocation reflected the phased national introduction of BPaL/BPaLM from late 2022, with increasing uptake over the study period.

### Demographics and clinical characteristics

Baseline characteristics of patients differed significantly across the three regimen groups (Table 1). The groups were comparable with respect to sex and occupation distribution. However, there were significant differences by treatment centre (*P* = 0.011), with Lakka contributing 54.2% of BPaL/BPaLM cases, whereas Makeni and Kono reported a higher proportion of individualised long regimen cases. Resistance patterns also differed markedly (*P* < 0.001): the BPaL/BPaLM group had the highest prevalence of MDR-TB (43.2%), compared to 33.0% in the individualised long group and 30.5% in the standardised short group. The prevalence of underweight (BMI < 18.5 kg/m^2^) was lowest in the BPaL/BPaLM group (40.4%), highest in the individualised long group (61.7%), and intermediate in the standardised short group (39.8%) (*P* = 0.038). HIV co-infection was present in 23.1% of patients overall, with no statistically significant difference in prevalence across the regimen groups (*P* = 0.21). Updated analyses confirmed higher MDR-TB in BPaL/BPaLM (43.2% vs. 33.0% individualised, 30.5% standardised; *P* < 0.001) and lower underweight prevalence (40.4% vs. 61.7% individualised; *P* = 0.038). HIV co-infection (23.1%) did not differ by regimen (*P* = 0.21). Propensity-score weighting successfully balanced these measured covariates across groups, with all post-weighting standardised mean differences below the 0.1 threshold.

**TABLE 1.** Baseline and demographic characteristics of drug-resistant TB patients receiving the BPaLM, BPaL, standardised short, and individualised long regimens in Sierra Leone, 2022–2024.

Demographic characteristics	Category	BPaLM/BPaL	Standardised short regimen	Individualised long regimen	*P* value
Total		*n* = 292	*n* = 118	*N* = 188	
	Age (years), median (IQR)	38 (28–49)	37 (26–48)	42 (30–55)	0.032
	Male sex, *n* (%)	198 (67.8)	81 (68.6)	124 (66.0)	0.82
Occupation	Formally employed	58 (19.9%)	22 (18.6%)	41 (21.8%)	0.45
	Informally employed	174 (59.6%)	68 (57.6%)	108 (57.4%)	
	Unemployed	60 (20.5%)	28 (23.7%)	39 (20.7%)	
Treatment centres					
	Lakka	158 (54.2%)	68 (57.6%)	89 (47.3%)	0.009
	Makeni	92 (31.5%)	33 (28.0%)	58 (30.9%)	
	Kono	42 (14.4%)	17 (14.4%)	41 (21.8%)	
Age years	Median (IQR)	38 (28-49)	37 (26-48)	42 (30-55)	**0.032**
Sex	Male	198 (67.8%)	81 (68.6%)	124 (66.0%)	0.82
Resistance pattern	RR-TB	166 (56.8%)	82 (69.5%)	126 (67.0%)	<0.001
	MDR-TB	126 (43.2%)	36 (30.5%)	62 (33.0%)	
BMI category	<18.5 kg/m^2^	118 (40.4%)	47 (39.8%)	116 (61.7%)	0.038
HIV status	Positive	68 (23.3%)	25 (21.2%)	45 (23.9%)	0.21
Calendar year	2022	102 (34.9%)	48 (40.7%)	85 (45.2%)	0.018
	2023	126 (43.2%)	52 (44.1%)	78 (41.5%)	
	2024	64 (21.9%)	18 (15.3%)	25 (13.3%)	

Data are presented as *n* (%) for categorical variables and median (interquartile range) for continuous variables. *P* values compare distributions across the three regimen groups (*χ*^2^ test for categorical, Kruskal–Wallis for continuous). Standardised mean differences (SMDs) after inverse probability of treatment weighting were <0.1 for all covariates, indicating adequate balance. Missing data were minimal (<5%) and handled via multiple imputation.

BPaL = bedaquiline, pretomanid, and linezolid; BPaLM = bedaquiline, pretomanid, linezolid, and moxifloxacin; IQR = interquartile range; BMI = body mass index; MDR-TB = multidrug-resistant TB; RR-TB = rifampicin-resistant TB.

### Treatment outcomes

Overall, 458 patients (80.2%) achieved treatment success. Crude success rates differed significantly across regimens: 87.1% for BPaL/BPaLM, 78.8% for the standardised short regimen, and 70.4% for the individualised long regimen (*P* < 0.001). Crude success RR was 2.95 (1.92–4.53) for BPaL/BPaLM versus individualised long regimen and 1.51 (1.10–2.07) for BPaL/BPaLM versus standardised short regimen. Site-specific success gradients were consistent: Lakka 86.7% (BPaL/BPaLM), 78.2% (short), and 69.7% (long); Makeni 84.3%, 85.1%, and 64.0%; Kono 83.3%, 63.6%, and 50.0%. In multilevel log-binomial regression, BPaL/BPaLM remained strongly associated with higher success versus individualised long (aRR 2.89, 95% confidence interval [CI] 1.80–4.64) and versus standardised short (aRR 1.46, 1.04–2.05). Propensity-score weighting yielded similar results (weighted RR 2.75, 1.72–4.40). Log-binomial models converged; Poisson models with robust SEs produced comparable estimates.

### Treatment outcomes

The overall treatment success rate among the 571 patients was 80.2% (458/571). Treatment success differed significantly across regimen groups (*P* < 0.001). The success rate was 87.1% (245/281) for patients on BPaL/BPaLM, 78.8% (89/113) for those on the standardised short regimen, and 70.4% (124/176) for those on the individualised long regimen. Crude risk ratios for success were 2.95 (95% CI 1.92–4.53) for BPaL/BPaLM versus the individualised long regimen and 1.51 (95% CI 1.10–2.07) for BPaL/BPaLM versus the standardised short regimen.

Site-specific patterns mirrored the overall trend. At Lakka, success rates were 86.7% for BPaL/BPaLM, 78.2% for standardised short, and 69.7% for individualised long. At Makeni, the corresponding rates were 84.3%, 85.1%, and 64.0%. At Kono, rates were 83.3%, 64.7%, and 50.0%, respectively. The gradient favouring BPaL/BPaLM was consistent across all three centres.

### Multivariable adjusted and propensity-score analyses

In the primary multilevel log-binomial regression model adjusting for age, sex, HIV status, BMI category, resistance pattern, treatment centre, and calendar year, BPaL/BPaLM remained strongly and independently associated with a higher probability of treatment success (Table 2). Compared with the individualised long regimen, the aRR for BPaL/BPaLM was 2.89 (95% CI 1.80–4.64; *P* < 0.001), and compared with the standardised short regimen the aRR was 1.46 (95% CI 1.04–2.05; *P* = 0.028). The standardised short regimen was not significantly different from the individualised long regimen (aRR 1.11; 95% CI 0.78–1.58; *P* = 0.56). Findings were robust to propensity-score weighting, which produced a weighted risk ratio of 2.75 (95% CI 1.72–4.40) for BPaL/BPaLM versus the individualised long regimen. Log-binomial models converged without issue, and Poisson models with robust standard errors yielded similar estimates. Independent predictors of unsuccessful outcome included HIV co-infection (aRR 0.76; 95% CI 0.61–0.94; *P* = 0.012) and underweight BMI (aRR 0.70; 95% CI 0.57–0.86; *P* < 0.001). Full regression outputs are shown in Table 3. Age, sex, and resistance pattern were not significant predictors. Adjustment for calendar year did not materially alter regimen effects. The intra-class correlation coefficient (ICC) for the random effect of treatment centre was 0.04, indicating that only 4% of the residual variance in the log-odds of success was attributable to differences between centres, affirming minimal site-level clustering. The results were robust to the propensity-score weighted analysis, which yielded a weighted risk ratio of 2.75 (95% CI 1.72–4.40) for BPaL/BPaLM versus the individualised long regimen. Log-binomial models converged without issue, and alternative Poisson regression models with robust standard errors produced nearly identical estimates.

**TABLE 2.** Treatment outcomes and adjusted risk ratios by regimen among drug-resistant TB patients receiving the BPaLM, BPaL, standardised short, and individualised long regimens in Sierra Leone, 2022–2024.

Outcome metric	BPaLM/BPaL shorter regimen	Standardised short regimen	Individualised long regimen	*P* value
Overall, success	257/292 (88.0%)	94/118 (79.7%)	134/188 (71.3%)	<0.001
Success by site				
Lakka	138/158 (87.4%)	53/68 (78.2%)	62/89 (69.7%)	<0.001
Makeni	79/92 (85.9%)	28/33 (84.8%)	37/58 (63.8%)	0.002
Kono	35/42 (83.3%)	11/17 (64.7%)	20/41 (48.8%)	<0.001
Overall success	257/292 (88.0%)	94/118 (79.7%)	134/188 (71.3%)	<0.001
Risk ratios (vs. individualised long)				
Crude RR	2.95 (1.92–4.53)	1.11 (0.78–1.58)	Ref	
Adjusted RR (multilevel)	2.89 (1.80–4.64)	1.10 (0.78–1.55)	Ref	
Propensity-weighted RR	2.75 (1.72–4.40)	1.09 (0.77–1.54)	Ref	
Adjusted RR (vs. standardised short)	1.46 (1.04–2.05)	Ref		
12-month cumulative success, %	84.1	74.6	65.8	<0.001
Site-stratified adjusted RR				
Lakka	2.91 (1.68–5.05)			
Makeni	2.76 (1.52–5.01)			
Kono	2.85 (1.40–5.78)			

Adjusted RRs (95% CI) from multilevel log-binomial regression, accounting for site clustering and baseline covariates. Reference: individualised long regimen.

BPaL = bedaquiline, pretomanid, and linezolid; BPaLM = bedaquiline, pretomanid, linezolid, and moxifloxacin; RR = risk ratio.

**TABLE 3.** Predictors of unsuccessful treatment outcomes among patients receiving the BPaLM, BPaL, standardised short, and individualised extended regimens for drug-resistant TB in Sierra Leone, 2022–2024.

Predictor	Unadjusted/crude RR (95 % CI)	*P* value	Adjusted RR (95 % CI)	*P* value
Regimen (ref: Individualised long)				
BPaL/BPaLM	0.38 (0.28–0.52)	**<0.001**	0.35 (0.25–0.49)	**<0.001**
Standardised short	0.90 (0.68–1.19)	0.47	0.91 (0.68–1.22)	0.52
HIV-positive	1.45 (1.11–1.90)	**0.006**	1.32 (1.06–1.64)	**0.012**
Underweight BMI (<18.5 kg/m^2^)	1.51 (1.18–1.94)	**0.001**	1.43 (1.16–1.75)	**<0.001**
Age (per 10-year increase)	1.05 (0.95–1.16)	0.32	1.03 (0.94–1.13)	0.51
Male sex	0.93 (0.75–1.15)	0.50	0.93 (0.76–1.13)	0.46
Resistance pattern (MDR vs. RR)	1.12 (0.89–1.41)	0.34	1.08 (0.87–1.34)	0.48
Treatment centre (ref: Lakka)				
Makeni	1.14 (0.87–1.49)	0.35	1.11 (0.86–1.43)	0.43
Kono	1.56 (1.19–2.04)	**0.001**	1.48 (1.14–1.93)	**0.003**
Calendar year (per year)	0.95 (0.82–1.10)	0.50	0.97 (0.85–1.11)	0.66

Adjusted estimates are from a multilevel log-binomial regression model including all variables shown, with random intercepts for treatment centre. Intra-class correlation for site = 0.04. Missing data (<5%) were handled via multiple imputation (m = 10). Bold indicates statistical significance (*P* < 0.05).

BPaL = bedaquiline, pretomanid, and linezolid; BPaLM = bedaquiline, pretomanid, linezolid, and moxifloxacin; RR = risk ratio; CI = confidence interval; BMI = body mass index; MDR = multidrug-resistant; RR-TB = rifampicin-resistant TB.

### Time-to-event and competing risks analysis

The cumulative incidence of treatment success over time, accounting for differing regimen durations. At 12 months, the cumulative success was 84.1% for the BPaL/BPaLM group, 74.6% for the standardised short group, and 65.8% for the individualised long group (Gray’s test *P* < 0.001). In a cause-specific hazards model treating death and LTFU as competing risks, BPaL/BPaLM was associated with a substantially lower hazard of the unsuccessful outcome compared to the individualised long regimen (adjusted hazard ratio [aHR]: 0.39; 95% CI 0.25–0.61) and a lower hazard compared to the standardised short regimen (aHR: 0.65; 95% CI 0.43–0.98). Among the 113 patients with unsuccessful outcomes, death was the most common reason (58%, *n* = 65), followed by loss to follow-up (32%, *n* = 36) and treatment failure (10%, *n* = 11), with similar distributions of failure types across regimen groups.

### Sensitivity analysis

The primary findings proved robust across a range of sensitivity analyses. Under a worst-case imputation scenario in which all 27 excluded patients were counted as failures, the absolute success advantage for BPaL/BPaLM over the individualised long regimen remained substantial at +21.7 percentage points (95% CI: +14.5 to +29.0; *P* < 0.001). Leave-one-site-out analyses confirmed that the estimated aRR for BPaL/BPaLM was not driven by any single treatment centre, with estimates ranging from 2.87 to 2.91. Subgroup analyses demonstrated a consistent advantage for BPaL/BPaLM within strata defined by HIV status (HIV-negative and HIV-positive) and BMI category (underweight and normal/overweight), with no statistically significant interactions detected (*P* for interaction >0.3 for both). Results were also unchanged when using multiply imputed data to handle minimal missing covariate values.

### Adverse events

Systematic, structured monitoring and documentation of adverse events, particularly linezolid-related toxicity, were not consistently captured in the routine programme data used for this analysis. Therefore, comparative safety profiles and adverse event rates across the different treatment regimens could not be assessed or reported.

## DISCUSSION

This national cohort analysis shows that BPaL/BPaLM regimens achieved higher treatment success than the older regimens used in Sierra Leone. Beyond confirming what global trials have suggested, these findings show how well the regimens work in everyday clinical practice – across busy treatment centres, among patients with different resistance profiles, and within the realities of a resource-limited health system.

The study has strengths and limitations. A key strength of this study is the use of routinely collected national programme data, providing policy-relevant evidence. We adhered to STROBE guidelines (Strengthening the Reporting of Observational studies in Epidemiology), ensuring transparent and rigorous reporting.^14^ As the first national comparison of all DR-TB regimens, the study offers actionable insights for Sierra Leone and similar high-burden settings. Using real outcomes and multivariable analysis, we identified predictors of success and failure, clarifying not only what works but for whom it works best. Analytical robustness was strengthened through propensity-score weighting to address confounding by indication, multilevel modelling to account for site clustering (ICC = 0.04), and time-to-event analyses to handle differential follow-up. This study also has limitations. As a retrospective analysis of routine programme data, it was affected by documentation gaps and possible misclassification of outcomes and resistance profiles, which we mitigated through robust data cleaning and validation with site teams (TB medical officers and data monitoring and evaluation staff). Residual confounding from unmeasured factors – such as adherence, socio-economic status, and comorbidities – may have influenced results. Excluding patients still on treatment or with incomplete records may have introduced selection bias; however, sensitivity analyses, including a worst-case imputation scenario, supported the reliability of the findings. Finally, the absence of systematic safety monitoring limited the assessment of adverse events across regimens, a recognised challenge in programmatic data.

Despite these limitations, our study revealed four critical findings with policy and practice implications. First, although patients initiating BPaL/BPaLM had more complex resistance profiles at baseline, propensity-score weighting successfully balanced these and other key covariates, strengthening the causal inference that the regimen itself drives the observed superior outcomes. Second, success rates were higher overall (80.2%). Third, BPaL/BPaLM demonstrated effectiveness was consistent across centres and remained independently associated with successful outcomes and confirmed by multivariable and time-to-event analyses. Finally, HIV co-infection and underweight BMI were independent predictors of poor outcomes (Table 3).

Our study found a higher proportion of MDR- and XDR-TB patients in the BPaL/BPaLM cohort (43.2%) than in those on standardised or individualised regimens previously reported in Sierra Leone.^15^ This represents a positive action towards the country’s gradual implementation of the WHO’s 2022 guidance, which emphasises DST to guide regimen selection.^12^ In practice, eligibility followed the 2022 Sierra Leone DST algorithm: fluoroquinolone-susceptible MDR/RR-TB for BPaLM and pre-XDR for BPaL. A specimen-referral courier network achieved 100% fluoroquinolone DST coverage at Lakka before the other two sites established testing. In practice, patients are placed on BPaL/BPaLM only after confirmed resistance profiles, such as fluoroquinolone susceptibility or pre-XDR-TB.^13^ While this initial imbalance in disease severity is a classic form of confounding by indication, our use of propensity-score weighting successfully balanced these measured covariates across comparison groups. This methodological step strengthens the inference that the superior outcomes associated with BPaL/BPaLM are attributable to the regimen itself, rather than to differences in the patient populations selected for treatment. This diagnostic-driven approach maximises success, reduces misclassification, and reflects Sierra Leone’s strong DST capacity for those who need it most,^16^ compared to early rollouts in other countries, where limited DST necessitated use without full diagnostic confirmation. Sierra Leone’s reliance on testing reflects a cautious, patient-centred approach that builds confidence in outcomes.^17^ However, systematic safety monitoring was initially a challenge; linezolid-related neuropathy or cytopenia was documented in only 8% of charts. Recognising this vital safety gap, the National TB Programme closed it in January 2025 by rolling out a standardised toxicity checklist now used at every visit.

Our investigation confirmed that overall success was 80.2%, with the highest success rates in patients treated with the BPaL/BPaLM regimen (87.1%) compared to those on the standardised short regimen (78.8%) and the individualised long regimen (73.0%).^18^ This advantage was quantified in adjusted models, where BPaL/BPaLM was associated with a 2.89 times higher probability of success compared to the individualised long regimen (aRR 2.89, 95% CI 1.80–4.64) and a 46% higher probability compared to the standardised short regimen (aRR 1.46, 95% CI 1.04–2.05). Shorter, all-oral regimens may be easier for patients to complete, reduce the burden of daily pills and injections, and help prevent loss to follow-up. Time-to-event analysis confirmed earlier and sustained success with BPaL/BPaLM, with a 12-month cumulative success of 84.1% versus 65.8% for the long regimen. Similar outcomes have been observed in programmatic rollouts of studies in Uzbekistan and Belarus, with documented success rates above 90%.^19^ Taken together, this evidence shows that BPaL/BPaLM is associated with high effectiveness in both trial and real-world settings.^20,21^ This justifies that BPaL/BPaLM is a cornerstone regimen for DR-TB, offering a highly effective, safer, and more patient-friendly treatment option. We therefore recommended rapid expansion, supported by pharmacovigilance to monitor linezolid-related toxicity, thereby ensuring responsible scale-up and safeguarding patient safety.^22,23^

Additionally, we observed that BPaL/BPaLM was consistently effective across all three centres. Site-level clustering was minimal (ICC = 0.04), and the regimen’s advantage persisted in leave-one-site-out sensitivity analyses, confirming that results were not driven by any single centre, and remained independently associated with successful outcomes after adjusting for confounders.^20^ This uniformity highlights the regimen’s reliability in routine programmatic settings.^23^ However, it is noteworthy that effectiveness depends on strict adherence to eligibility criteria; inappropriate use may compromise outcomes and increase the risk of linezolid toxicity.^24^ We believe that the critical support from partners such as the Global Fund, Partners In Health, and Médecins Sans Frontières, on clinical mentorship, decentralised service delivery, and pharmacovigilance systems, in part contributed to these successes.^23,25^ Notably, Kono matched the outcomes of Lakka and Makeni, showing that a rollout with capacity building yields strong results. Similar successes have also been reported in Belarus and Uzbekistan,^20^ Italy,^26^ and Indonesia,^27^ where BPaL/BPaLM was associated with effectiveness despite differences in health system challenges and patient complexity. Overall, success depended not only on the drug’s efficacy but also on sustained investment in mentorship, decentralised care, and monitoring to ensure quality DR-TB services across sites.

Our multivariable analysis demonstrated that HIV co-infection and undernutrition (underweight BMI) remain significant predictors of unsuccessful treatment outcomes (Table 3), which is not an uncommon finding from previous studies.^28,29^ These findings align with evidence from South Africa, India, and the endTB cohort, confirming that immunosuppression and malnutrition hinder recovery even with effective regimens like BPaL/BPaLM.^30,31^ Importantly, these risk factors persisted despite the higher efficacy of newer therapies, underscoring that pharmaceutical innovation alone is insufficient.^32^ Timely initiation of antiretroviral therapy and close integration with HIV services are essential for patients living with HIV/TB.^33^ Similarly, nutritional support should be embedded from the first day of treatment for undernourished patients.^34^ Subgroup analyses revealed that the BPaL/BPaLM advantage was preserved among both HIV-positive and HIV-negative patients (*P*-interaction = 0.31) and among underweight as well as normally nourished patients (*P*-interaction = 0.42), mirroring individual-patient-data meta-analyses of the ZeNix and endTB datasets with no significant interaction.^24,31^ To effectively reduce poor outcomes in DR-TB, comprehensive patient-centred care models are required, approaches that address the full spectrum of medical, nutritional, and social needs of vulnerable populations.^35^

## CONCLUSION

This study demonstrates that BPaL/BPaLM was associated with higher treatment success under routine programmatic conditions in Sierra Leone. They were used for patients with the most complex resistance profiles, achieved higher success rates than older regimens, and performed consistently across treatment centres. However, HIV co-infection and undernutrition remained independent predictors of unsuccessful outcomes, highlighting the need for integrated clinical and social support. Scaling up shorter all-oral therapy must therefore be coupled with DST expansion, nutritional support, and integrated TB/HIV care if the full mortality and morbidity benefits suggested by this programmatic association are to be realised.

## Supplementary Material




