# Supplementary material for: Evaluating treatment outcomes stratified by regimen among drug-resistant TB patients in Sierra Leone
Source: Public Health Action. 2026 Mar 6;16(1):28–34. doi: 10.5588/pha.25.0056 (PMC12991776; doi:10.5588/pha.25.0056)
Supplement: Supplementary file 1 [file pha25-0056_supplementarydata1.pdf]

Supplementary Table 1: Propensity score balance diagnostics

Covariate	Unweighted SMD	Weighted SMD (IPTW)	Balance Achieved?
**Regimen (ref: Individualised long)**			
Age (per 10 years)	0.22	0.04	Yes
Male sex	0.03	0.02	Yes
HIV-positive	0.12	0.03	Yes
Underweight (BMI <18.5)	0.31	0.06	Yes
MDR-TB (vs. RR-TB)	0.25	0.05	Yes
Treatment centre (ref: Lakka)	0.18	0.05	Yes
Calendar year 2023	0.15	0.04	Yes
Calendar year 2024	0.10	0.03	Yes
Age (per 10 years)	0.22	0.04	Yes
Male sex	0.03	0.02	Yes
HIV-positive	0.12	0.03	Yes
Underweight (BMI <18.5)	0.31	0.06	Yes

SMD = Standardized Mean Difference; IPTW = Inverse Probability of Treatment Weighting. Threshold for balance: SMD <0.1.

Supplementary table 2: sensitivity analyses – robustness check

Analysis	BPaL/BPaLM vs. Individualised Long (aRR, 95% CI)	BPaL/BPaLM vs. Standardised Short (aRR, 95% CI)
Primary Model (Multilevel)	2.89 (1.80–4.64)	1.46 (1.04–2.05)
Complete-case (n=571)	2.85 (1.78–4.57)	1.44 (1.03–2.02)
Multiple Imputation (m=10)	2.89 (1.80–4.64)	1.46 (1.04–2.05)
Poisson with Robust SEs	2.91 (1.81–4.68)	1.47 (1.05–2.06)
Worst-case Imputation	2.91 (1.82–4.66)	1.47 (1.05–2.06)
Leave-One-Site-Out:		
• Excluding Lakka	2.87 (1.65–5.00)	1.45 (1.03–2.04)
• Excluding Makeni	2.90 (1.70–4.95)	1.46 (1.04–2.05)
• Excluding Kono	2.88 (1.75–4.73)	1.46 (1.04–2.05)
Analysis	BPaL/BPaLM vs. Individualised Long (aRR, 95% CI)	BPaL/BPaLM vs. Standardised Short (aRR, 95% CI)
Primary Model (Multilevel)	2.89 (1.80–4.64)	1.46 (1.04–2.05)
Complete-case (n=571)	2.85 (1.78–4.57)	1.44 (1.03–2.02)
Multiple Imputation (m=10)	2.89 (1.80–4.64)	1.46 (1.04–2.05)

Supplementary table 3: Subgroup analyses

Subgroup	BPaL/BPaLM Success	Standardised Short Success	Individualised Long Success
HIV Status			
• HIV-negative	90.2% (202/224)	81.4% (83/102)	73.1% (104/142)
• HIV-positive	80.9% (55/68)	72.0% (18/25)	64.4% (29/45)
BMI Category			
• Normal/Overweight (≥18.5)	91.4% (159/174)	84.5% (60/71)	76.4% (55/72)
• Underweight (<18.5)	83.1% (98/118)	72.3% (34/47)	68.1% (79/116)
Treatment Centre			
• Lakka	87.4% (138/158)	78.2% (53/68)	69.7% (62/89)
• Makeni	85.9% (79/92)	84.8% (28/33)	63.8% (37/58)
• Kono	83.3% (35/42)	64.7% (11/17)	48.8% (20/41)

Supplementary table 4: competing risks analysis – cause-specific hazards

Outcome Type	BPaL/BPaLM vs. Individualised Long (aHR, 95% CI)	BPaL/BPaLM vs. Standardised Short (aHR, 95% CI)
**Death**	0.42 (0.26–0.68)	0.70 (0.45–1.09)
**Loss to Follow-up**	0.35 (0.20–0.61)	0.58 (0.35–0.96)
**Treatment Failure**	0.48 (0.25–0.92)	0.75 (0.40–1.41)

Adjusted for age, sex, HIV, BMI, resistance, centre, and calendar year. aHR = Adjusted Hazard Ratio

Supplementary table 5: missing data handling

Variable	Missing, n (%)		Handling Method
BMI category	22 (3.7%)		Multiple Imputation
HIV status	14 (2.3%)		Multiple Imputation
Resistance pattern	9 (1.5%)		Multiple Imputation
Prior TB	18 (3.0%)		Multiple Imputation
Occupation	25 (4.2%)		Multiple Imputation
Variable	Missing, n (%)	Handling Method	Imputation Model

All variables imputed using chained equations (m=10 imputations). Convergence confirmed with trace plots.

Supplementary table 6: Calendar time adjustment impact

Model Specification	BPaL/BPaLM vs. Individualised Long (aRR, 95% CI)	ICC for Centre
Without calendar year	2.88 (1.79–4.63)	0.04
With calendar year (primary)	2.89 (1.80–4.64)	0.04
With calendar year as categorical	2.89 (1.80–4.64)	0.04

Minimal change in effect estimates with calendar time adjustment.
